# Dogs and cats as sources of companionship and emotional support in vulnerable animal caregivers

**DOI:** 10.3389/fpsyg.2026.1831814

**Published:** 2026-07-08

**Authors:** Mónica Boada, Thaïs Sánchez, Noe Terrassa, Antoni Bulbena, Jonathan Bowen, Jaume Fatjó

**Affiliations:** 1 Affinity Foundation Chair for Animals and Health, Department of Psychiatry and Forensic Medicine, School of Medicine, Autonomous University of Barcelona, Barcelona, Spain; 2 Foundation for Advice and Action in the Defence of Animals (FAADA), Barcelona, Spain; 3 Queen Mother Hospital for Small Animals, Royal Veterinary College, North Mymms, United Kingdom

**Keywords:** companion animals, dog-human relationship, human-animal interaction, social health, social support, vulnerable populations

## Abstract

Social support may be crucial for the health and wellbeing of populations facing adversity, such as homelessness, intimate partner violence, or economic vulnerability. However, vulnerable individuals are usually at a high risk of social exclusion, having restricted social networks and limited social support. Companion animals constitute a potential source of support, but their relative contribution compared to other sources remains underexplored. In this study, we adopted a multifaceted approach to social support to investigate the relative importance and supportive functions of human and animal sources within the social support networks of 100 vulnerable individuals who were users of an intervention program. Participants rated the degree to which they considered their closest companion animal (dog/cat) a family member and answered a questionnaire regarding their social support network, in which they nominated and ranked sources of support based on their relevance. Our findings demonstrate that dogs and cats were considered genuine family members and constituted an important source of support. They were frequently the sole providers of companionship and emotional comfort, as well as of opportunities for physical closeness and nurturance. However, our results also indicate that human relationships remain necessary to meet other aspects of social support, such as obtaining guidance (informational support), receiving material or behavioural assistance (instrumental support) and gaining social recognition or approval. In sum, companion animals are a significant part of the already fragile social networks of vulnerable individuals; hence, protecting this bond is fundamental.

## 1 Introduction

Social support refers to the availability of assistance from others in one’s social network, and entails the provision of resources that help individuals cope with stressful life events. It can thus be essential for populations facing highly adverse situations, such as people experiencing homelessness, victims of intimate partner violence, or economically vulnerable older adults. Numerous studies highlight the associations between social support and connections and better health and coping outcomes in vulnerable populations, suggesting a potential protective effect against the negative consequences of adversity (Coker et al., 2002, 2003; Beeble et al., 2009; Dickens et al., 2011; Chen et al., 2014; Cummings et al., 2022; Mah et al., 2022). However, individuals from these populations are usually at a high risk of social exclusion, often having restricted social networks and limited social support (Zugazaga, 2008; Wilson et al., 2011; Katerndahl et al., 2013; Watson et al., 2016; Donovan and Blazer, 2020; Faraji and Metz, 2021; Nolet et al., 2021; Cummings et al., 2022). Hence, investigating and enhancing the availability of social support for vulnerable populations might be fundamental for improving their health and wellbeing.

Sources of social support usually comprise individuals within one’s social network with whom one shares close relationships. Although these typically include romantic partners, family and friends, a potential source of social support that is often overlooked is companion animals. In Europe, it is estimated that there are around 352 million companion animals, with the most common being cats and dogs (FEDIAF, 2024). In a global survey across five continents and ten countries, 94% of caregivers agreed that their companion animal was part of their family, and 89% agreed that they shared a close relationship (HABRI, 2023). Likewise, when thinking about their companion animals, “family member” and “like a child” were the most common descriptors used by caregivers. Correspondingly, there is an increasing shift away from traditional anthropocentric views of family systems and toward a broader recognition of multispecies families (Irvine and Cilia, 2017; Applebaum et al., 2021). Viewing companion animals as family members is associated with greater perceptions of socially supportive traits in these animals and improves wellbeing (McConnell et al., 2019). An expanding body of literature confirms that companion animals serve as significant sources of social support for their caregivers (McConnell et al., 2011, 2016; Bowen et al., 2020, 2021; Fatjó and Bowen, 2023; Hardie et al., 2023; Liu et al., 2024; Sudbury-Riley, 2024; Turcsán et al., 2025). Consequently, not accounting for companion animals as potential sources of support could result in an incomplete understanding of individuals’ support networks.

Research on vulnerable populations shows that, as in the general population, companion animals provide social support, and that the potential protective effects of social support in adversity are not exclusive to human sources (Gee and Mueller, 2019; Kerman et al., 2019; Krause-Parello et al., 2019; Cleary et al., 2020, 2021; Hughes et al., 2020; Applebaum et al., 2021; Reniers et al., 2023). Importantly, the social support provided by companion animals might possess unique attributes that enhance its significance within these populations. One of the possible advantages of companion animals as sources of support is their increased availability compared to human sources. Availability refers to how easily a source of social support can be accessed (Fatjó and Bowen, 2023). We have previously found that most caregivers perceive their dogs as highly available sources of support to which they can readily turn during a crisis (Bowen et al., 2021). This aspect is especially relevant for individuals at risk of social exclusion, for whom companion animals might constitute the only consistent and reliable source of emotional support (Flynn, 2000b; Fitzgerald, 2007; Cleary et al., 2020; Reniers et al., 2023). Moreover, companion animals are often regarded as non-judgmental and providers of unconditional love (Flynn, 2000a; Fitzgerald, 2007; Labrecque and Walsh, 2011; Newberry, 2017; Hageman et al., 2018; Kerman et al., 2019; Scanlon et al., 2021; Reniers et al., 2023), which, again, can be particularly meaningful for individuals from vulnerable populations, who frequently feel stigmatized (Coker et al., 2002; Petrovich and Cronley, 2015).

Crucially, sources of support differ in the functions they serve (Thoits, 2011). While companion animals may be particularly suited to provide certain types of social support and might substitute human sources for some functions when they are unavailable (Bowen et al., 2021), other kinds of support can only be provided by humans. Thus, it is important to characterize the functional role that companion animals play in the social support network. The social support literature is characterized by considerable conceptual heterogeneity, with no universally accepted taxonomy (for a review see Thomas and Hodges, 2026). Core dimensions of social support include emotional support, social contact and companionship, information and guidance support, and instrumental/tangible support. *Emotional support* represents a diverse category with multiple manifestations, including providing opportunities for self-disclosure and showing caring behaviours, such as demonstrations of affection (affectionate support), comforting touch, enhancement of self-worth or provision of social approval/recognition (esteem support) and unconditional availability (“being there for someone”). Relatedly, it has been proposed that one of the main provisions of social relationships is the opportunity for nurturance or caregiving (Weiss, 1974; Furman and Buhrmester, 1985; Turcsán et al., 2025), and that social support promotes thriving, for example by helping individuals find a purpose in life (Feeney and Collins, 2015). *Social contact and companionship support* relates to the availability of partners to participate in social and leisure activities. *Information and guidance support* refers to the provision of facts or advice. *Instrumental or tangible support* entails the provision of behavioural or material assistance.

Companion animals mainly contribute through emotional and social contact/companionship, whereas informational and instrumental forms of support are limited to specific contexts involving specially trained animals, such as assistance or working dogs (Gravrok et al., 2020). Companion animals may generate a sense of purpose and mattering, increase self-esteem and foster a sense of achievement, provide a sense of belonging, and act as stress-buffers (Fatjó and Bowen, 2023). We have previously identified five features of the social support caregivers can get from companion animals: availability, shared activities, physical contact, self-disclosure and opportunities for caregiving (Bowen et al., 2021; Fatjó and Bowen, 2023). It is worth mentioning that companion animals may also provide social support indirectly by facilitating social interactions between people that lead to the formation of supportive relationships (Wood et al., 2015). Yet, the support offered directly by companion animals must still be complemented with that provided by humans to satisfy all social needs.

While previous research with vulnerable populations has established that companion animals constitute a source of support, their relative contribution compared to other sources across multiple aspects of social support remains underexplored. The present study tries to address this gap in the literature and complements prior work examining this topic in the general population. Some of these earlier studies were based on attachment theory and investigated the role of companion animals as attachment figures relative to human relationships. Attachment is closely related with social support at the conceptual level, as it involves close affectional bonds that provide emotional and companionship support (Meehan et al., 2017). Four attachment features are usually examined: (1) proximity seeking/maintenance (2) secure base, (3) safe haven and (4) separation distress. Attachment figures are used as sources of support, and support-seeking behaviour can be considered a manifestation of the attachment behavioural system (Collins and Feeney, 2000); consequently, attachment measures typically allude to social support aspects. Research shows that companion animals, especially dogs, are included in attachment hierarchies and can surpass human figures on certain attachment features (Kurdek, 2008, 2009a, 2009b; Meehan et al., 2017). Proximity maintenance and separation distress were the most salient attachment features in dogs (Kurdek, 2009a, 2009b), confirming that they are especially valued for their companionship. Interestingly, although safe haven was dogs’ least salient attachment feature, in times of emotional distress, people were more likely to turn to their dogs than to their parents, siblings, children and best friends (Kurdek, 2009a, 2009b). These results prove that dogs also play an important role in the provision of emotional comfort. Apart from attachment theory, social provisions theory has been adopted to compare people’s relationship with their companion animals to their relationship with humans across several scales, which covered multiple social support aspects. Dogs received higher scores than human relationships, including closest kin, romantic partners and best friends, on scales measuring companionship, nurturance (i.e., opportunities for caregiving), affection, reassurance of worth and reliability (Turcsán et al., 2025). Overall, these findings highlight the relevance of companion animals in the fulfilment of attachment-related functions and social support needs, and demonstrate how comparing relationships with companion animals to various types of human relationships across different facets can provide deeper insight into their role within the social network.

In this study, we investigated the role of companion animals as a source of support for individuals in vulnerable situations who were users of an intervention program. We aimed to confirm the hypothesis that companion animals are considered family members by these individuals, and to explore the supportive functions animals fulfilled within their (limited) social support network. We adopted a multifaceted approach to social support, recognizing that it is not a unitary construct but encompasses several aspects, such as sharing activities, receiving comfort, having a confidant, gaining approval and having caregiving opportunities, among others. By asking the participants to nominate and rank their sources of support in the order they would turn to them to fulfil specific needs, or according to the perceived degree to which each source satisfied those needs, we could determine the composition of the social support network, as well as the functions served by and the relative importance of human and animal sources of support. We also examined associations between animal and human sources of support to explore whether companion animals complement human sources (complement hypothesis) or compensate for limited human support (compensatory/hydraulic hypothesis) (McConnell et al., 2011).

## 2 Methods

### 2.1 Ethical note

This study was reviewed and approved by the Ethical Commission on Animal and Human Experimentation (CEEAH) of the Autonomous University of Barcelona (UAB) (#5794).

### 2.2 Sample

The sample was comprised of 100 adults in a verified situation of vulnerability that had been living with one or more companion animals for over 6 months and were users of the Best Friends (“Millors Amics/Mejores Amigos”) program of the Foundation for Advice and Action in the Defence of Animals (FAADA). This program is aimed at users of public social services who have a positive and non-exploitative/instrumentalist bond with their companion animal, including people experiencing homelessness, victims of intimate partner violence and older adults with compromised social networks and/or precarious living conditions, among others. Its primary goal is to raise awareness and protect the human-animal bond within vulnerable populations. Its main actions are supporting social services in case management, offering specific education and tools, facilitating access to shelter services with animals, and providing free veterinary care in verified situations of vulnerability.

The sociodemographic characteristics of the sample can be found in Supplementary Table S1. Participants were between 19 and 80 years old (*M* = 48.5, *SD* = 12.5), with 60% identifying as female, 39% as male and 1% as transgender. Although all participants were highly vulnerable, their profiles were diverse, including individuals experiencing homelessness, inadequate housing, economic deprivation, single-mother families, older adults, victims of intimate partner violence, people with physical or mental health conditions, and individuals falling into multiple categories. The most common profiles were individuals experiencing homelessness (*n* = 30), victims of intimate partner violence (*n* = 21) and older adults (*n* = 19).

Most participants only had dogs (79%), followed by both dogs and cats (12%) and only cats (9%). Regarding the number of animals, the majority of participants had only one dog (59%), followed by two dogs (14%), and one dog and one cat (6%), with other combinations reported less frequently.

### 2.3 Procedure

Upon admission to the Best Friends program, users were asked whether they would like to participate in an anonymous interview exploring human-companion animal relationships. Those who agreed were contacted and interviewed in person or online later that year. First, the demographic information of participants (i.e., age and gender) was registered, as well as whether they lived with dogs and/or cats, and the number of dogs/cats they had. Next, the participant was asked to rate the degree to which they considered their closest companion animal a family member. Following this, the participant answered a series of questions regarding their social support network. Interviews were conducted by trained interviewers who assisted participants when necessary (T.S. or N.T.) and usually lasted approximately 15 min.

### 2.4 Measures

#### 2.4.1 Family membership scale

Participants were asked to indicate where they would place their closest companion animal on a scale of 1 to 10 based on the degree to which they considered them a family member (1 = the animal is not a family member, 10 = the animal is a full-fledged family member).

#### 2.4.2 Social support network questionnaire

Our exploration of the participants’ perceived social support network was based on previous approaches to the study of both human and animal attachment figures and sources of social support (Rowe and Carnelley, 2005; Tancredy and Fraley, 2006; Kurdek, 2009b; Meehan et al., 2017; Bowen et al., 2021). We conceptualized social support as a multifaceted construct comprising at least the following aspects: sharing activities, experiencing companionship, engaging in physical contact, receiving comfort, getting advice, obtaining assistance, having a confidant, gaining approval and having caregiving opportunities. We developed a questionnaire in Spanish drawing from instruments such as the WHOTO Questionnaire (Hazan et al., 1991; Hazan and Zeifman, 1994, as cited by Gillath et al., 2016; see also Fraley and Davis, 1997) and the Monash Dog-Owner Relationship Scale (MDORS; Dwyer et al., 2006), to obtain hierarchies of sources of support (Trinke and Bartholomew, 1997).

The questionnaire consisted of 19 items, which covered the aforementioned social support aspects. Participants were asked to nominate and rank individuals according to the order they would turn to them in different situations (e.g., “Who do you turn to first to share that you have achieved something good?”), or according to the degree to which they fulfilled each aspect (e.g., “Who is always there when you need them?”). Participants were reminded that their answers could include both animals and humans and were instructed to assign individuals to five ranked positions. They were allowed to nominate more than one individual in each rank. Participants were instructed to nominate only sources they considered genuinely relevant and were allowed to leave ranks empty or items unanswered.

An English version of the questionnaire can be found in Table 1. Items 1–6 were taken from the WHOTO questionnaire (Hazan et al., 1991; Hazan and Zeifman, 1994, as cited by Gillath et al., 2016), with each pair of items measuring an attachment-related function/feature in the original conceptualization (items 1–2: proximity-seeking, items 3–4: safe haven, items 5–6: secure base). From a social support perspective, items 1–2 can be interpreted as addressing companionship, item 3 emotional comfort, item 4 informational support, item 5 social approval/recognition, and item 6 touches on support availability or reliability. The rest of items were derived from the MDORS (Dwyer et al., 2006) and had been previously used to assess aspects related to perceived social support from companion animals, including: stated support (items 7–8), availability (items 9–12), shared activities (items 13–14), self-disclosure (item 15), physical contact (items 16–17) and opportunities for caregiving (items 18–19) (Bowen et al., 2021). From an attachment theory perspective, attachment and social support may operate at different conceptual levels rather than representing fully independent constructs. Attachment processes organize expectations regarding proximity, emotional availability, and security, whereas social support reflects the interpersonal and behavioural expression of these relational systems in everyday life and stressful situations. In this sense, support-seeking and caregiving behaviours may be conceptualized as manifestations of attachment-related systems rather than entirely separate phenomena. This perspective may help explain why measures of emotional social support frequently overlap with constructs such as attachment security, companionship, and perceived availability (Collins and Feeney, 2000; see also Feeney and Collins, 2015).

**Table 1:** Social support network questionnaire.

Item
1. Who do you enjoy spending your time with?
2. Who do you not like being away from?
3. Who do you like to be with when you are upset or feeling down about something?
4. Who do you value the most for advice?
5. Who do you turn to first to share that you have achieved something good?
6. Who can you always count on?
7. Who helps you the most in difficult times?
8. Which individuals give you reasons to get up every morning?
9. If everyone else abandoned you, who do you think would always be there?
10. Who do you think keeps you company the most?
11. Who is always there when you need them?
12. Who is more attentive to you?
13. Who do you share the most activities or fun moments with?
14. Who do you spend the most time with while resting?
15. Who do you tell things you would not share with anyone else?
16. Who do you hug most often?
17. Who do you kiss most often?
18. Who do you give gifts to most often?
19. Who do you enjoy taking care of the most?

### 2.5 Data analysis

Data collected between 2020 and 2024 were included in the analysis. We conducted descriptive analyses to summarize and extract information from the raw data in Microsoft Excel and Jamovi (version 2.6.44.0, The Jamovi Project, 2025).

As our focus was on support provided by members of participants’ social network, only responses referring to identifiable living social agents (i.e., humans or animals capable of direct interaction with the participant) were retained. Answers referring to supernatural entities, deceased individuals, objects, activities, or abstract concepts were excluded, as these are not conventionally considered members of a personal social network. These included God (mentioned by *n* = 4 subjects), deceased humans or animals (*n* = 4), volunteering (*n* = 2), the Bible (*n* = 1), a book (*n* = 1), a plant (*n* = 1), the internet (*n* = 1) and life (*n* = 1). Responses where the participant referred to themselves were also removed. If deleting a response created an empty rank but the item still contained other responses, the remaining responses were reassigned to ranks in their original order, ensuring all rank positions were filled contiguously. As responses referring to groups of individuals (e.g., “dogs,” “friends,” “children”) were accepted during data collection, analysing the exact number of individuals nominated was not feasible. We therefore focused on source types instead and standardized plural labels to their singular form (e.g., “friends” to “friend,” “children” to “child”). Moreover, because we were not interested in gender differences of the sources, labels were transformed to gender-neutral forms (e.g., son/daughter to “child,” father/mother to “parent,” brother/sister to “sibling,” nephew/niece to “nibling,” uncle/aunt to “pibling”). Pre-processed data can be found in Supplementary Table S2.

Variables derived from the social support network questionnaire are briefly described in Table 2 and are explained in more detail below. Data were analysed at both the subject and item levels (see Supplementary Tables S3, S4).

**Table 2:** Description of variables from the social support network questionnaire.

Subject-level (across items)	
Number of questions with no source of support	
Number of questions in which CA were nominated	
Number of questions in which CA were the only source	
Number of source types	For each subject, number of different source types nominated in the questionnaire
Top source	For each subject, most frequently nominated source in the questionnaire
Number of top source mentions	For each subject, number of questions in which the top source was nominated
Overall rank (for each source type)	For each subject, average of the rank of each source type across items. If the source type was nominated more than once in an item, the lowest numerical rank was taken.
Proportion of questions CA top rank	For each subject, number of items in which companion animals were ranked first (excluding those where they were the only source) divided by the number of items in which companion animals were not the only source

Item-level (across subjects)	
Number of subjects with no source of support	
Number of subjects who nominated CA	
Number of subjects with CA as only source	
Mean rank (for each source type)	For each item, average of the rank of each source type across subjects. If the source type was nominated more than once by a subject, we took the lowest numerical rank.
Proportion of subjects CA top rank	For each item, number of subjects who ranked companion animals first (when they were not the sole source of support) divided by the number of subjects who nominated companion animals alongside other sources in that item

CA, companion animals.

*Subject-level*. For each participant, we counted the number of different source types (e.g., “dog,” “child,” “parent”) they nominated across the questionnaire. It should be noted that, as mentioned above, this number does not represent the exact number of individual sources of support. We also counted the number of items in which each source was nominated to determine the most frequently nominated source (i.e., “top source”). Additionally, for each participant we recorded the number of items in which they did not report any source, the number of items in which companion animals were nominated, and the number of items in which they were the only source of support. For source types of interest (companion animals and five human relationships, see below), we identified the highest position that source type was assigned in each item where it was nominated (i.e., lowest numerical rank) and then averaged these values across items to obtain an overall rank for that source type for each participant. Finally, we calculated the proportion of items in which companion animals were ranked first. Because companion animals necessarily received the top rank when they were the only source of support, we excluded those items. The proportion was therefore computed as the number of items in which companion animals were ranked first and were not the sole source, divided by the total number of items in which companion animals were not the only source.

*Item-level*. For each item, we recorded the number of subjects who did not report any source, the number of subjects who nominated companion animals, and the number of subjects who mentioned them as their only source. For source types of interest, we identified the highest position they were assigned by each subject in each item (i.e., lowest numerical rank) and then averaged these values across subjects to obtain a mean rank for each item. Lastly, we calculated the proportion of subjects who ranked companion animals first, dividing the number of subjects who ranked them first when they were not the sole source of support by the total number of subjects who mentioned companion animals alongside other sources.

To characterize the overall distribution of the variables, we computed summary statistics, mainly based on the subject-level data, which can be found in Supplementary Table S5.

In addition, we compared companion animals with five human relationships as sources of support. Because human figures were infrequently nominated at the item level, we focused on overall ranks. Analyses included only participants who provided ranks for both companion animals and the relevant human figure at least once across the questionnaire. Pairwise comparisons with parents, children, siblings, friends and romantic partners were conducted. Because the variables were mean ranks derived from ordinal data, and since the distribution of differences did not consistently meet the assumption of normality (Shapiro–Wilk test and histogram and Q–Q plot inspection), we used Wilcoxon signed-rank tests for all pairwise comparisons.

Moreover, we explored associations between animal and human social support. Specifically, we examined the correlation between the number of human source types reported and the number of questions in which companion animals were nominated, the number of questions in which they were the only source, the proportion of questions in which they were ranked first and their overall rank. We also explored associations between demographic variables (gender and age) and social support variables. Since most variables violated normality assumptions (Shapiro–Wilk test), we used Mann–Whitney U tests to assess gender differences (between males and females) and Spearman’s rank correlation to examine associations.

For all analyses, the significance level was set at 0.05 and *p*-values were adjusted for multiple comparisons using Holm’s method. Analyses were performed in RStudio (version 2025.09.2.418, Posit Team, 2025).

## 3 Results

### 3.1 Companion animals as family members

Regarding participants’ consideration of their companion animal as a family member, the mean score in the scale was 9.96 ± 0.2 (for all descriptive statistics see Supplementary Table S5). A value lower than 10 was only indicated by five participants, of which four gave a score of 9 and one of 9.5.

### 3.2 Social support network

#### 3.2.1 Composition

Support sources included, among others, informal supporters such as companion animals, friends, immediate family (parents, siblings and children), extended family (grandparents, grandchildren, uncles/aunts, cousins, nephews/nieces), romantic partners, neighbours/roommates, as well as formal supporters such as social services (e.g., social workers, educators) and health professionals (e.g., psychologists). Participants nominated between two and ten different types of sources. The mean number of source types was 4.22 ± 1.64. Thirty-five percent of participants did not report any source of support in at least one of the items, most frequently in items referring to obtaining advice (*n* = 15), approval (*n* = 13) and self-disclosure (*n* = 12).

#### 3.2.2 The role of companion animals

##### 3.2.2.1 Nomination frequency

Among all sources, companion animals were nominated (i.e., were mentioned at least once) by the highest percentage of participants. While all participants included companion animals in their social support network, 68% mentioned friends, 67% immediate family (which included parents, siblings and children), 37% extended family (which included grandparents, grandchildren, uncles/aunts, cousins, nephews/nieces and unspecific answers “relative” and “family”), 30% social services (which included social workers, educators, FAADA and community centres) and 20% romantic partners. Specifically, dogs were part of the social support network of 93% of participants, followed by friends (68%), children (37%), parents (36%), siblings (23%), cats (21%) and romantic partners (20%). All participants that had dogs cited them as sources of support, and among cat caregivers, only one did not mention their cat. Some participants nominated companion animals as sources of support even if they were not their caregiver (among non-dog caregivers, two nominated dogs, while among non-cat caregivers, one nominated a cat).

For 90% of participants the top source of support in the questionnaire (i.e., the source mentioned in the highest number of questions) was a companion animal (dog and/or cat). In two additional cases dogs were the top source of support along with a human source (child and parent, respectively). The mean number of questions in which companion animals were mentioned was 14.8 ± 2.81. For 98% of participants companion animals were the only source of support in at least one of the questions. The mean number of questions in which companion animals were the only source of support was 9.46 ± 4.7.

**Figure 1:**
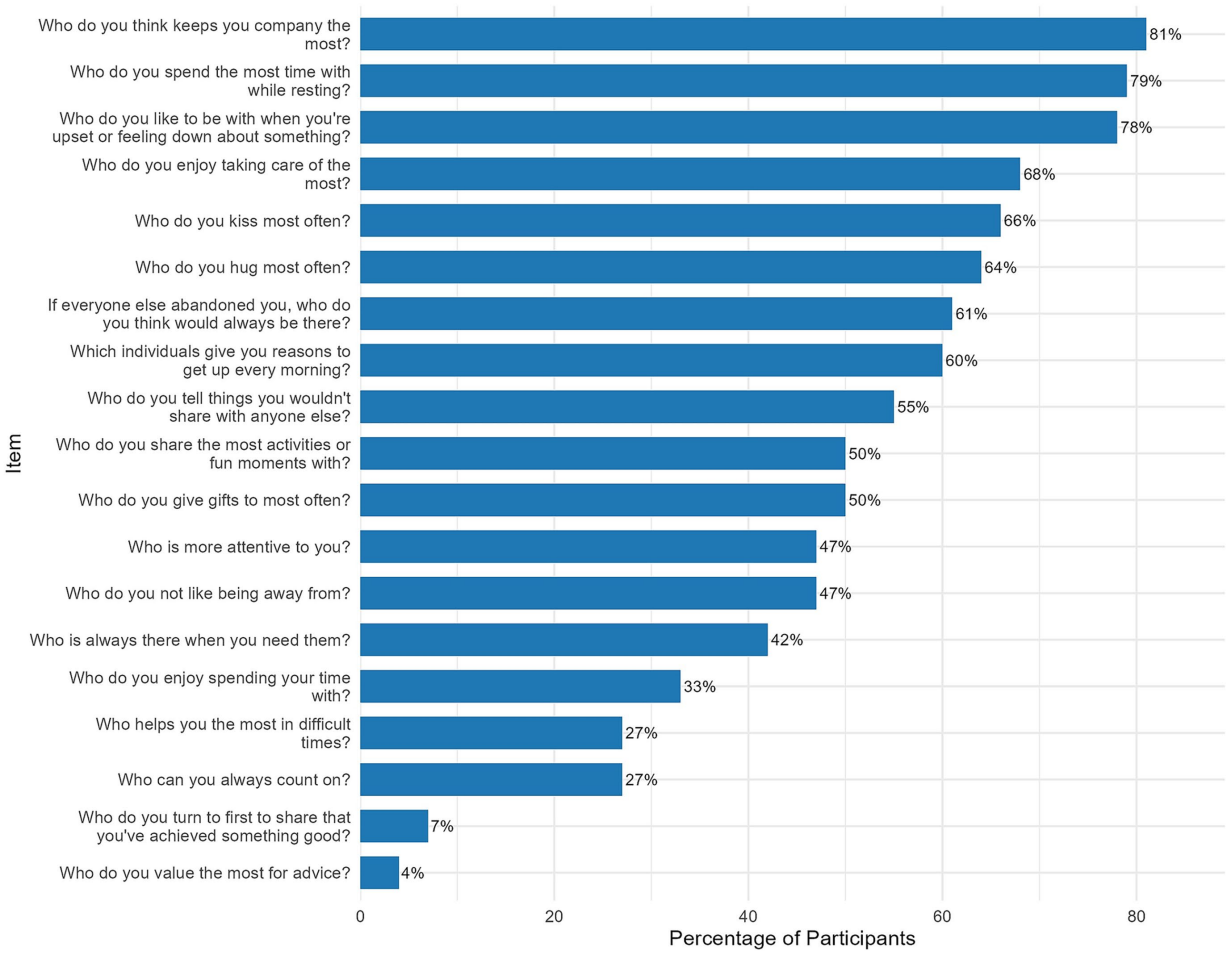
Percentage of participants who only nominated companion animals for each item.

Table 3 presents the number of participants who nominated companion animals and the most common human relationships in each item. The percentage of participants who nominated companion animals as their sole source of support in each item is shown in Figure 1. As can be observed, companion animals were mentioned by over 80% of participants in most items, surpassing human relationships in social support aspects such as companionship/shared activities, opportunities for caregiving, physical contact, emotional comfort and availability. Likewise, they were frequently cited as the only source of support in items related with these aspects. In contrast, they were seldom nominated in items that referred to provision of advice –where they were surpassed by friends, parents, children and romantic partners– and social approval –where they were surpassed by friends, children and parents–. Indeed, dogs were the most frequently nominated source of support in all items, except for these two, where friends emerged as the most nominated source.

**Table 3:** Item-level frequency of nominations for companion animals and human relationships.

Item	N					
	Companion animals	Friends	Children	Parents	Siblings	Romantic partners
Who do you think keeps you company the most?	99 (81)	5	6	1	2	4
Who do you enjoy taking care of the most?	98 (68)	4	16	4	1	7
Who do you enjoy spending your time with?	97 (33)	40	26	5	3	11
Who do you spend the most time with while resting?	97 (79)	3	5	3	1	10
Who do you not like being away from?	94 (47)	8	26	11	4	9
Who do you hug most often?	94 (64)	3	15	2	2	7
Which individuals give you reasons to get up every morning?	90 (60)	5	20	4	1	7
Who do you like to be with when you are upset or feeling down about something?	89 (78)	4	8	0	1	1
If everyone else abandoned you, who do you think would always be there?	89 (61)	9	10	11	9	5
Who do you kiss most often?	89 (66)	2	15	2	1	6
Who do you share the most activities or fun moments with?	85 (50)	20	14	2	0	8
Who is more attentive to you?	84 (47)	14	15	14	5	7
Who is always there when you need them?	83 (42)	20	16	13	8	7
Who do you tell things you would not share with anyone else?	72 (55)	11	10	3	3	6
Who do you give gifts to most often?	72 (50)	6	25	5	1	4
Who can you always count on?	68 (27)	28	18	16	12	10
Who helps you the most in difficult times?	56 (27)	21	15	10	5	10
Who do you turn to first to share that you have achieved something good?	16 (7)	27	23	19	7	10
Who do you value the most for advice?	5 (4)	34	9	11	5	8

*N* represents the number of participants who nominated each source in each item. The number of participants who nominated companion animals as their only source is in parenthesis. Items are presented in descending order based on the frequency of nomination of companion animals.

##### 3.2.2.2 Ranking

**Figure 2:**
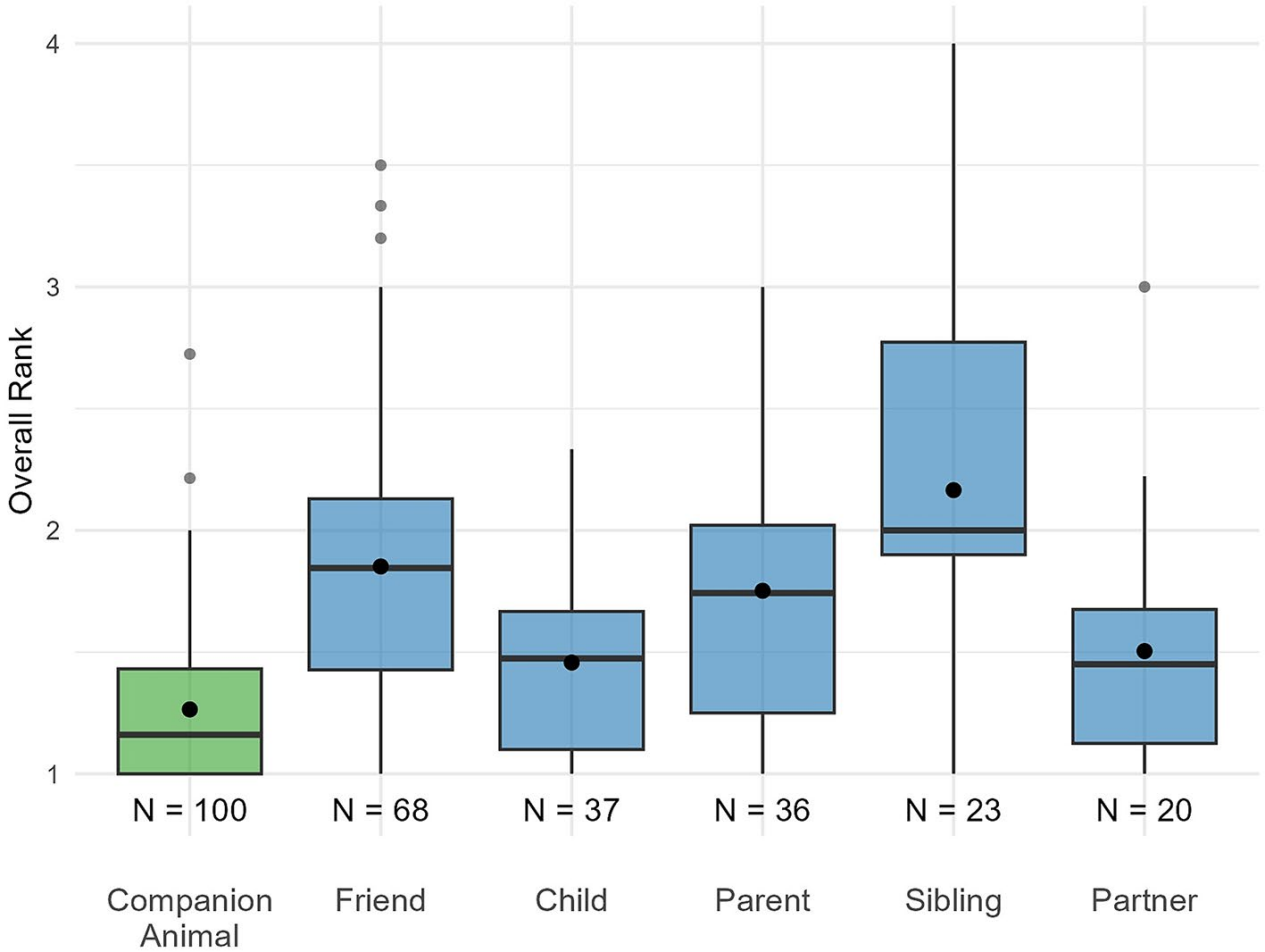
Overall rank of companion animals and five human relationships. Boxplots show the medians (thick horizontal line), means (black dot), interquartile ranges (box), data range (vertical lines) and outliers (grey dots). N indicates the number of participants who nominated each source at least once. Lower values indicate a higher position in the ranking.

The position of companion animals within the hierarchy of the social support network ranged from first to fifth. The overall ranks for companion animals and the most common human relationships are shown in Figure 2. The mean overall rank for companion animals across subjects was 1.26 ± 0.3 (for all descriptive statistics see Supplementary Table S5). Item-level data can be found in Supplementary Table S4.

Results of the Wilcoxon signed rank tests comparing the overall ranks of companion animals to five human relationships are presented in Table 4. These analyses included only participants who provided ranks for both companion animals and the relevant human figure at least once across the questionnaire. There was a significant difference between companion animals and friends, parents and siblings, with companion animals being ranked higher overall (i.e., lower rank scores), but no difference with children and romantic partners.

**Table 4:** Wilcoxon signed rank test results.

Relationship	*N*	Median (IQR) companion animal	Median (IQR) human relationship	*z-*value	*p*-value	Adjusted *p*-value	Effect size (*r*)
Friend	**68**	**1.16 (0.37)**	**1.85 (0.7)**	**−5.69**	**<0.001**	**<0.001**	**0.69**
Parent	**36**	**1.27 (0.42)**	**1.74 (0.77)**	**−3.09**	**0.002**	**0.006**	**0.52**
Child	37	1.44 (0.46)	1.47 (0.57)	−0.02	0.988	0.988	0.002
Sibling	**23**	**1.18 (0.42)**	**2.00 (0.87)**	**−3.85**	**<0.001**	**<0.001**	**0.80**
Partner	20	1.29 (0.33)	1.45 (0.55)	−0.86	0.391	0.781	0.19

Significant differences based on adjusted *p*-values are in bold.

Keeping only questions where companion animals were nominated and were not the only source of support, the mean proportion of questions in which they were ranked first was 0.57 ± 0.35. Keeping only participants who mentioned companion animals together with other sources, the mean proportion of participants who ranked animals first was 0.6 ± 0.2. Excluding cases where companion animals shared the first rank with other sources only changed these proportions slightly (mean proportion of questions = 0.54 ± 0.36, mean proportion of participants = 0.55 ± 0.2).

**Figure 3:**
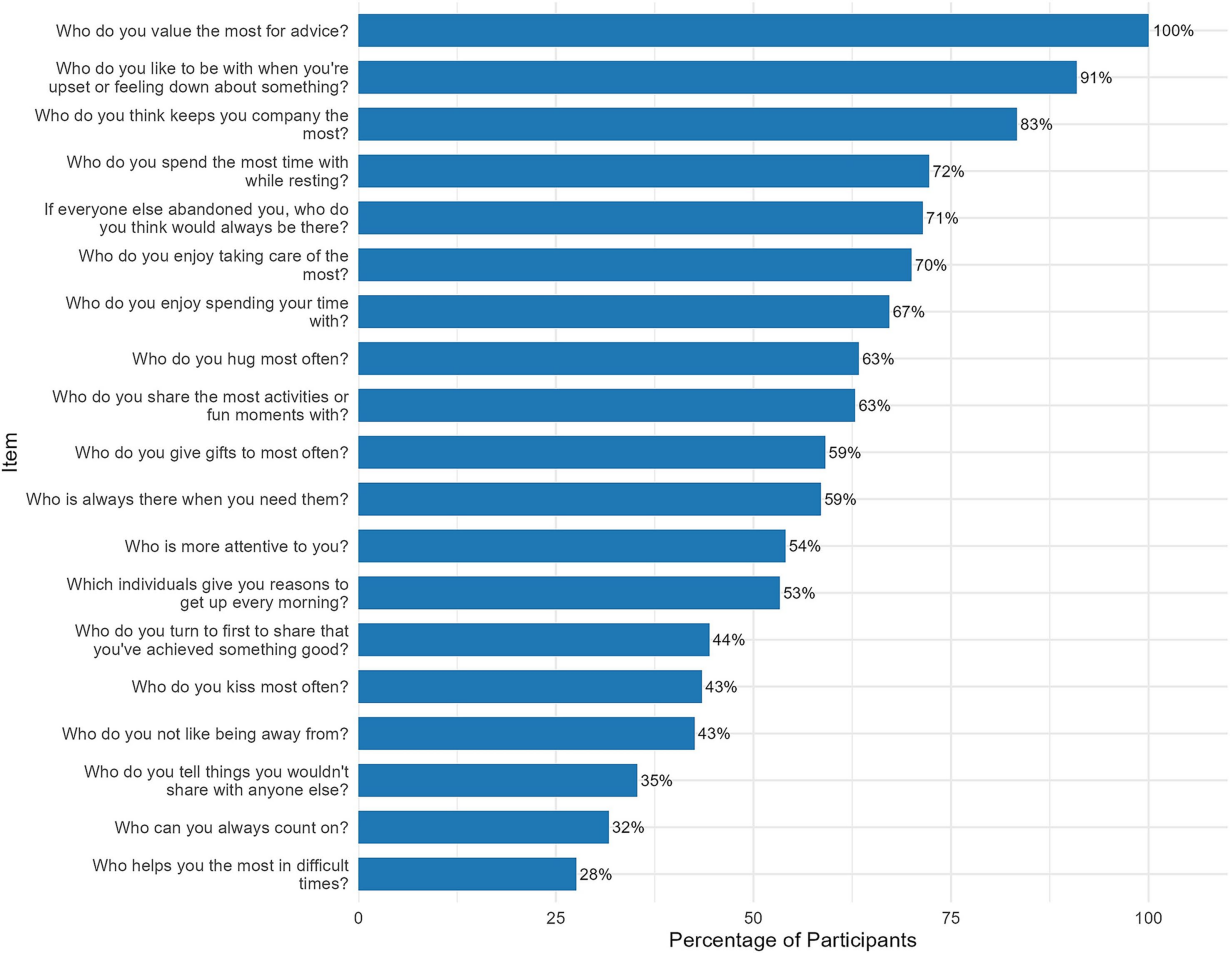
Percentage of participants who ranked companion animals first for each item. Proportions are relative to the number of participants who nominated companion animals in each item. Cases where companion animals were the only source of support were excluded.

Figure 3 shows, for each item, the percentage of participants who ranked a companion animal as their first source of support. This proportion was calculated dividing the number of subjects who ranked companion animals first by the total number of subjects who nominated companion animals for each item, excluding cases where animals were the only source of support (see Table 5). Note that for many items the number of subjects who nominated companion animals is small because most participants nominated them as the only source (see Table 3). These results should be interpreted with this caveat in mind. In most cases companion animals were the only source nominated in rank 1, only sharing the first rank with a human source in 28 cases. For most social support aspects examined, the majority of participants ranked their companion animal first. The items where animals were ranked first proportionally less frequently implied obtaining assistance, self-disclosure, separation distress, providing physical affection, and receiving approval, while they were ranked first proportionally more often in items related with receiving advice, emotional comfort and companionship.

**Table 5:** Number of participants who nominated and ranked companion animals first in each item (excluding cases where they were the only source).

Item	Number of participants who…	
	Nominated CA	Ranked CA first
Who do you think keeps you company the most?	18	15 (1)
Who do you enjoy taking care of the most?	30	21 (3)
Who do you enjoy spending your time with?	64	43 (2)
Who do you spend the most time with while resting?	18	13 (2)
Who do you not like being away from?	47	20 (1)
Who do you hug most often?	30	19 (3)
Which individuals give you reasons to get up every morning?	30	16 (2)
Who do you like to be with when you are upset or feeling down about something?	11	10
If everyone else abandoned you, who do you think would always be there?	28	20 (1)
Who do you kiss most often?	23	10 (2)
Who do you share the most activities or fun moments with?	35	22 (3)
Who is more attentive to you?	37	20 (2)
Who is always there when you need them?	41	24 (1)
Who do you tell things you would not share with anyone else?	17	6
Who do you give gifts to most often?	22	13 (3)
Who can you always count on?	41	13 (1)
Who helps you the most in difficult times?	29	8 (1)
Who do you turn to first to share that you have achieved something good?	9	4
Who do you value the most for advice?	1	1

Items are presented in descending order based on the frequency of nomination of companion animals. Cases where companion animals were the only source of support are excluded. Cases where companion animals shared the first rank with other sources are in parenthesis. CA, companion animals.

##### 3.2.2.3 Associations with human support

We examined the relationship between the number of human source types and the role of companion animals in the support network. We found a significant negative correlation with the number of items in which companion animals were the only source (*r*_s_ (98) = −0.34, adjusted *p*-value = 0.002). In addition, there was a significant correlation with the overall rank of companion animals (*r*_s_ (98) = 0.3, adjusted *p*-value = 0.008), indicating that participants who nominated more human source types assigned companion animals a lower position (i.e., a higher numerical rank). Because the overall rank of companion animals is directly related to the number of items in which they were the only source –as they necessarily received the top rank in those cases– we computed a partial correlation. After controlling for the number of items in which companion animals were the only source, the correlation between the number of human source types and the overall rank of companion animals was no longer significant (*r*_s_ (98) = 0.09, *p*-value = 0.382). The proportion of questions in which animals were ranked first –which excluded items in which they were the sole source– was not significantly correlated with the number of human relationship types. While we found a negative correlation with the number of items in which companion animals were nominated, it did not remain statistically significant after adjustment for multiple comparisons. All correlations are reported in Supplementary Table S6.

#### 3.2.3 Demographic analyses

We found a significant gender difference in the number of source types and the number of questions in which companion animals were the only source, with males reporting fewer source types and more frequently nominating animals as their sole source compared with females. However, these gender differences did not remain statistically significant after adjustment for multiple comparisons. Group descriptive statistics and test results are reported in Supplementary Table S7. Regarding age, we found a significant positive correlation with the number of questions in which companion animals were the only source (*r*_s_ (98) = 0.3, adjusted *p*-value = 0.016); that is, older individuals nominated animals as their sole source more often than younger individuals. All correlations are reported in Supplementary Table S8.

## 4 Discussion

To the best of our knowledge, this is the first study to directly compare the relative importance and supportive functions of human and companion animal sources within the social support networks of vulnerable individuals. Our results provide evidence that dogs and cats are considered full-fledged family members and constitute an important source of support for vulnerable individuals, especially as providers of companionship and emotional support. However, our findings also denote the need for human connections to obtain other types of support, such as tangible resources and guidance for overcoming challenging situations.

### 4.1 Social support network composition

Participants’ social support networks included both informal supporters, like companion animals, friends, family, romantic partners and neighbours/roommates, as well as formal supporters such as social workers/services and health professionals. Participants nominated between two and ten different types of sources, with an average of four types of relationships. In this study, we did not measure the exact number of individual sources of support. Importantly, however, we did observe that approximately one out of three participants lacked support in at least one context, most often reporting they had no one from whom to seek advice, with whom to share their achievements or in whom to confide. This may indicate that some participants were not meeting basic social needs, which compounds their vulnerability. Our results align with previous research reporting restricted social networks and limited social support in vulnerable populations (e.g., Zugazaga, 2008; Katerndahl et al., 2013; Watson et al., 2016).

### 4.2 Companion animals as family members

Companion animals were viewed as genuine family members, as indicated by the uniformly high scores and the resulting ceiling effect in the scale. This finding adds to the accumulating evidence from both the general and vulnerable populations that companion animals are integrated into the family unit (e.g., Flynn, 2000a; Fitzgerald, 2007; Hageman et al., 2018; Cryer et al., 2021; Scanlon et al., 2021; HABRI, 2023). This might have important practical implications, given that individuals from vulnerable populations commonly face barriers and challenges associated with animal companionship (Toohey et al., 2017; Hageman et al., 2018; Kerman et al., 2019; Cleary et al., 2020, 2021; Scanlon et al., 2021; McCosker et al., 2024). Recognizing companion animals as part of the family unit may help to remove existing barriers and provide more effective assistance to vulnerable populations (Hageman et al., 2018; McCosker et al., 2024).

### 4.3 The supportive role of companion animals

#### 4.3.1 Nomination frequency

Companion animals played a major role in participants’ social support network, both at the structural and functional levels. Remarkably, companion animals were identified as sources of support by all participants, surpassing human sources such as friends, relatives and romantic partners. This result was largely attributable to dogs, which were part of the social support network of 93% of participants, representing the most common source (all participants who had dogs cited them as sources of support). This finding suggests that, for vulnerable individuals, companion animals may constitute a more available source of support than humans, possibly due to having limited human relationships or to a reduced reliance on them. Furthermore, companion animals were the source of support most frequently nominated across questions (i.e., the top source) for almost every participant, being mentioned in an average of 15 out of 19 questions. This means that most vulnerable individuals felt that companion animals provide them with support in more contexts than human figures. However, as the questionnaire focused especially on emotional and companionship support, this finding should be interpreted with this limitation in mind. Still, for nearly all participants companion animals were the only source of support in at least one of the studied aspects, supporting the relevance of preserving these relationships. Overall, these findings highlight the importance of considering companion animals as legitimate sources of support and the substantial support they may provide to vulnerable individuals.

#### 4.3.2 Ranking

Regarding the social support network hierarchy, companion animals often outranked humans and emerged as the main source of support across participants and questions. When participants nominated companion animals with other sources, they ranked animals first in an average of 54% of the questions. This implies that, despite participants having human sources of support, they were more likely to turn to companion animals in more than half of the situations, or they perceived animals as better fulfilling most social support needs, according to their self-reports. Likewise, on average, when companion animals were nominated among other sources in an item, over half of the participants placed animals at the top of the hierarchy. Thus, companion animals represented the primary source of support for numerous participants, even when they had multiple sources available to serve that function. As mentioned above, when interpreting these findings, it is important to take into account that most items addressed emotional and companionship support.

In addition, we found that participants who included companion animals in their social support networks alongside friends, parents or siblings, ranked animals higher overall, whereas no significant differences were observed when comparing companion animals with children or romantic partners. It should be noted that these analyses were exploratory; only participants who provided ranks for both companion animals and the relevant human figure at least once across the questionnaire were included, and ranks were averaged across items (see description of variables in Table 2). Our results align with previous studies in the general population showing that companion animals can surpass some human relationships in the fulfilment of certain social support needs (Bonas et al., 2000; Kurdek, 2008, 2009b, 2009a; Meehan et al., 2017; Turcsán et al., 2025) and suggest that this might also apply to vulnerable individuals.

#### 4.3.3 Associations with human support

Associations emerged between animal and human sources of support. Specifically, the role of companion animals within participants’ social support networks was related to the number of human relationship types reported. Our results showed that as individuals had access to a greater variety of human support sources, companion animals served as their sole of support in fewer contexts. Consequently, animals were assigned the highest position less frequently, thus having a lower overall rank. This pattern could reflect a compensatory dynamic within the support network, whereby animals become more important when human support is limited. At the same time, once companion animals were nominated with other sources, how often they represented the primary source of support (rank 1) did not depend on the number of human relationship types available. In other words, when human support is available, the number of contexts in which individuals turned to animals first or perceived them as better fulfilling a particular need was independent of the variety of human sources. This result provides support for the complement hypothesis, rather than the compensatory hypothesis, in line with studies in the general population (McConnell et al., 2011; Turcsán et al., 2025). However, it should be taken into account that we did not measure the exact number of individual sources of support.

Our findings raise the question of why relationships with companion animals became so central for many participants. One possibility, consistent with the recurring characterization of companion animals as non-judgmental and unconditionally accepting (Flynn, 2000a; Fitzgerald, 2007; Reniers et al., 2023), is that the human–animal bond may offer a form of affection comparatively free from the risks that can accompany strained human relationships—an attribute that may be especially salient for individuals whose human ties have been marked by adversity. Animals’ more limited communicative and evaluative capacities may paradoxically make them feel like safer recipients of self-disclosure (Evans-Wilday et al., 2018; Augustine and Eyssel, 2024). Such an interpretation would also be compatible with the suggestion that loneliness can heighten the attribution of social and supportive roles to companion animals (Epley et al., 2008), and with the broader observation that animals may compensate for limited human support in certain contexts while complementing it in others (McConnell et al., 2011; Turcsán et al., 2025). However, the present study was not designed to examine the developmental or psychological origins of these relationships, as we did not collect data on participants’ attachment histories or prior relational experiences. Object relations and attachment-based accounts offer promising interpretive frameworks for future research aiming to understand the affective centrality of these bonds.

#### 4.3.4 Functional aspects of social support

Analysing the individual aspects of social support assessed by the questionnaire yields a more nuanced understanding of the functional role of companion animals.

##### 4.3.4.1 Most salient social support aspects

In most social support aspects, including companionship/shared activities, opportunities for caregiving and for physical contact, emotional comfort and self-disclosure/intimacy, companion animals were nominated by at least 70% of participants.

*Companionship*. Almost every participant reported that animals kept them company the most, that they enjoyed spending time with them and that they did not like being away from them. This echoes earlier findings showing that proximity maintenance and separation distress were the most salient attachment features in the human-dog relationship (Kurdek, 2009b, 2009a) and that companionship is the most common reason for dog ownership (Jagoe and Serpell, 1996; Holland et al., 2022).

*Caregiving opportunities*. Similarly, nearly all participants stated that they enjoyed taking care of companion animals, which is in line with prior studies in which nurturance emerged as a particularly relevant facet of the human-dog relationship (Bonas et al., 2000; Turcsán et al., 2025).

*Physical contact*. Most participants also reported that they hugged and kissed companion animals often, suggesting that dogs and cats may be valuable sources of physical contact for vulnerable individuals, as observed in the general population (Bowen et al., 2021).

*Sense of purpose*. Remarkably, approximately 90% of participants considered that companion animals gave them reasons to get up every morning, which is consistent with previous research indicating that people –including vulnerable individuals such as older adults– derive a sense of purpose from animal companionship (Toohey et al., 2017; Bowen et al., 2021; Cryer et al., 2021), and that this serves as a motivation for acquiring a dog (Holland et al., 2022).

*Emotional comfort*. Furthermore, the vast majority of participants liked being with companion animals when emotionally distressed, assumably because they find their presence comforting. This concurs with existing literature showing that animals represent a significant source of emotional comfort (safe haven) (e.g., Kurdek, 2009b, 2009a; Meehan et al., 2017).

*Reliability*. Moreover, most participants thought that their companion animal would always be there even if everyone else abandoned them (item 9), which speaks to the perceived reliability of the bond, an aspect that has proved to be especially salient in the dog-human relationship (Bonas et al., 2000; Turcsán et al., 2025). While this item could be interpreted as implicitly excluding human sources (“If everyone else abandoned you.”), hence favouring companion animals, it should be noted that participants did nominate human sources in this item, sometimes exclusively. This implies that the potential bias introduced by the item wording did not affect participants uniformly.

Crucially, for many of these items, at least half of those who nominated companion animals reported them as their only source of support. Dogs and cats were most often the sole providers of companionship and emotional comfort, as well as of opportunities for physical closeness and nurturance. Nearly 80% of participants reported relying on companion animals exclusively in times of emotional distress. Our results therefore suggest that companion animals may be the only source of emotional support for vulnerable individuals, consistent with prior studies (Flynn, 2000b; Fitzgerald, 2007; Cleary et al., 2020; Reniers et al., 2023).

##### 4.3.4.2 Least salient social support aspects

The only items where companion animals had notably lower nomination frequencies were those involving seeking advice and social recognition, which require complex cognitive and communicative abilities. Provision of advice is a type of informational support that entails at least an understanding of the problem and the communication of potential courses of action, which is beyond the capacities of companion animals. Likewise, although companion animals can provide a sense of accomplishment through providing opportunities for caregiving (Labrecque and Walsh, 2011; Toohey et al., 2017; Fatjó and Bowen, 2023; Sudbury-Riley, 2024), they cannot express pride or recognize their caregivers’ achievements. Hence, it is surprising that some participants nominated companion animals as sources of support in these contexts.

*Advice-seeking*. In the case of provision of advice, out of the five participants who nominated dogs, four cited them as their only source of support. A potential interpretation of this result is that when human sources of support are limited, some individuals extend the range of social roles attributed to companion animals. In the literature, there is suggestive evidence that feelings of isolation or loneliness may be associated with ascribing anthropomorphic social traits to companion animals (Epley et al., 2008), although further research is needed. In our study, the perception of dogs as a source of advice is partly clarified by the response of one participant, who explained that he trusted his dog’s “instinct.” Another possible explanation for this result is that these participants were reluctant to report having no source, given that they provided an answer for all items.

*Social recognition*. In the case of social recognition, only approximately half of the participants who nominated dogs cited them as their only source; thus, a lack of human support cannot fully explain these results. This item referred to sharing one’s achievements, which is a form of self-disclosure. The valence and intimacy of the topics to be disclosed, as well as the psychological functions and risks of self-disclosure influence whom people choose as confidants (Evans-Wilday et al., 2018; Augustine and Eyssel, 2024). In our study, many participants reported disclosing to their companion animals things they would not share with anyone else (item 15), in line with previous research (Bowen et al., 2021). Disclosing something one would not tell anyone else may be motivated by distress relief, which is an intrapersonal motive that might be satisfied even without a recipient of self-disclosure (e.g., through expressive writing). In contrast, sharing achievements is typically linked to social approval, which represents an interpersonal motive that depends more strongly on human interaction. It is possible that the few participants who reported turning to animals to share their achievements in our study were seeking to fulfil intrapersonal motives, such as enhancing positive affect and reinforcing their sense of self-worth, rather than interpersonal ones. Interestingly, Turcsán et al. (2025) found that dogs were rated higher than closest kin and best friends in the scale “Reassurance of Worth,” though they cautioned that these results should be interpreted carefully due to the scale’s low reliability. Future research could examine the motives behind self-disclosure of different topics to companion animals.

##### 4.3.4.3 Social support aspects in relation to other sources

To explore the value of companion animals relative to other sources of support in particular contexts, we also examined, for each item, the proportion of participants who ranked companion animals first when they were not their sole source of support. These analyses were exploratory and proportions should be interpreted with caution, as they are relative to the number of participants who nominated companion animals together with other sources, which differed across items and were small in some cases (see Table 5).

In most items, over half of the participants ranked companion animals first. Remarkably, in times of emotional distress, 91% of participants preferred to turn to their companion animal than to other sources. Although this proportion represented a small number of participants (*n* = 10), it should be emphasized that this is because most participants who nominated companion animals in this item identified them as their sole source of support (*n* = 78).

Interestingly, the items “Who can you always count on?” (item 6) and “Who helps you the most in difficult times?” (item 7) emerged as aspects in which companion animals were less valued, with less than one third of participants ranking animals first. This result may reflect that while companion animals provide emotional comfort, helping others in times of need also implicates supplying behavioural or material assistance with practical problems, i.e., instrumental support (Thoits, 2011), for which human sources are necessary. A lower ranking in item 6 could also be indicative of a low perceived availability/reliability of the relationship. However, it should be noted that 71% of participants (*n* = 20) ranked companion animals first in the item “If everyone else abandoned you, who do you think would always be there?” which suggests that most individuals perceived their bond with their companion animal as the most reliable and enduring.

Notably, while nearly all participants disliked being separated from their companion animals, when they were nominated with human relationships, the majority of participants placed humans above animals. This might imply that separation from human relationships was more distressing for these participants than separation from companion animals.

Interestingly, most participants reported hugging companion animals more often than humans (item 16), whereas for kissing (item 17) the opposite pattern was found. One possible interpretation is that whom individuals choose as recipient depends on the nature of the physical contact, with most participants preferring to hug animals but to kiss humans. This is consistent with previous findings in the general population showing that hugging dogs was a more common physical interaction than kissing them (Bowen et al., 2021), which could be related to hygiene and/or safety concerns (Fatjó and Bowen, 2023). Alternatively, rather than indicating a preference, these results may reflect that companion animals are more available for physical contact, and this is only evident in hugging frequency due to the previously mentioned concerns associated with kissing animals. Another factor that might have influenced these results is social norms regarding physical affection towards different types of human relationships.

Similarly, although many participants stated using companion animals as confidants (item 15), often exclusively, when animals were nominated with other sources only approximately one third of participants placed them at the top of the hierarchy. This suggests that animals became less central as sources of support when participants considered they had other individuals to cover this social need, consistent with the compensatory hypothesis.

Of the few participants that reported sharing their achievements with companion animals among other individuals, nearly half turned to animals first. Perhaps these participants prioritized intrapersonal over interpersonal motives, or placed greater value on the lower risk associated with disclosing to companion animals, especially if the topic held a high degree of intimacy for them. Surprisingly, the only participant who nominated both animal and human relationships in the item addressing advice-seeking, ranked their dog above their friends, signalling that this individual might have a distorted view of their companion animal (see below).

### 4.4 Limitations

Several limitations may affect the interpretation and generalizability of our findings. One of the major limitations of the study is the sample size. Apart from the usual restrictions on generalizability, this meant that only 12 participants had both a dog and a cat. Therefore, we were unable to compare the kind of support people obtained from the two species. However, although the nature of human-animal interactions varies across species, some evidence suggests that the emotional dimension of the relationship may be relatively stable. In a study comparing matched samples of cat and dog owners, Howell et al. (2017) reported differences in owner-animal interaction patterns but found no significant differences in perceived emotional closeness. Since data was collected through self-report questionnaires, social desirability may have biased results. Participants might have exaggerated the extent to which they view their companion animal as family to enhance their image as caregivers. Although social desirability bias could have led participants to overstate social support availability in the questionnaire, we believe this is unlikely given that numerous participants openly admitted that they lacked support in at least one of the questions. Still, a pet-enhancement bias has been described, whereby caregivers hold a positively distorted view of their companion animals (El-Alayli et al., 2006). Another factor that may have influenced our results is the presence of order effects. Specifically, asking participants to rate the degree to which they considered their closest companion animal a family member might have accentuated the perception of socially supportive traits in these animals (McConnell et al., 2019), resulting in an inflation of their role in the subsequent social support network questionnaire. An additional consideration is the possibility of a sample selection bias, since participants were recruited through an intervention program specifically designed to support the human–animal bond in vulnerable populations. Potential users of the intervention program were pre-screened to ensure that the relationship they had with their companion animal was positive and not exploitative. Consequently, while participants’ strong views of companion animals as family members and social supporters probably reflect genuine sentiments, our sample may overrepresent individuals who maintain particularly close relationships with their companion animals.

Moreover, the prominent role of companion animals in the social support network observed in this study may raise some doubts. One concern is that their emergence as the most frequently mentioned source of support in terms of number of participants could simply reflect the fact that all participants had companion animals, whereas some might not have had comparable human relationships. Since we did not examine participants’ broader social networks, we cannot determine whether human relationships that were not mentioned in the questionnaire were not considered supportive or were simply absent. In response, we note that a key goal of the study was precisely to highlight that vulnerable individuals with companion animals may have limited support from human sources. Furthermore, participants were instructed to nominate only the most relevant sources, meaning that the mere existence of a relationship did not necessarily imply its inclusion in the social support network. Thus, the finding that most participants cited companion animals as sources of support cannot be attributed solely to the fact that all participants had them. Indeed, the fact that the questionnaire included aspects specific to human sources, namely provision of advice and social recognition, and that companion animals were indeed nominated less often and/or deemed less relevant for these, demonstrates that most participants distinguished the contexts in which animals can genuinely provide support, rather than mentioning them indiscriminately.

A second concern is that, although the questionnaire covered multiple aspects of social support, it emphasized certain types (e.g., emotional and companionship support) or aspects (e.g., opportunities for physical contact and caregiving) that are especially salient in human-animal relationships, potentially contributing to the prominence of companion animals across questions. Findings regarding nomination frequency in terms of number of questions should be interpreted with this caveat in mind. Notwithstanding this limitation, our findings show that in contexts where both animals and humans can provide social support, participants often turned to companion animals first or perceived animals as better fulfilling social needs.

Lastly, we did not collect detailed information about the participants themselves. Consequently, we cannot speculate on the psychological basis of the use of companion animals as sources of emotional support, which could originate in previous abandonment traumas or failures in human bonds.

### 4.5 Future directions

To address some of the limitations of our study, future research could map the composition of individuals’ social networks –including both humans and animals–, assess which network members are perceived as sources of support, and examine diverse aspects of social support to determine the specific social needs they fulfil. In addition, although it has been hypothesized that animals play a more prominent role in the support network of individuals facing vulnerability, comparative research with the general population is lacking. Studies directly comparing vulnerable and non-vulnerable populations would help clarify whether the patterns observed in the present study reflect characteristics of human–animal relationships more generally or are primarily associated with situations in which human networks are limited or difficult to access (e.g., Přibylová et al., 2025). Another potential future direction would be comparing vulnerable individuals who live with companion animals and those who do not, to better understand the specific role animals play within constrained support networks. Future research could also examine how the role of companion animals within support networks evolves over time. Longitudinal studies would help determine whether animals become more central as human networks shrink, or whether they occupy stable and complementary roles regardless of changes in social circumstances. Further research should explore the psychological processes underlying the attribution of social roles to companion animals. In particular, it would be valuable to investigate how individuals interpret and respond to animals’ behaviour when fulfilling different supportive functions, and whether limitations in human support networks are associated with broader social attributions toward companion animals. Futures studies could also examine potential differences between companion animal species, such as cats and dogs, in the style and quality of emotional social support they provide. Although challenging, it would be very valuable to collect detailed information about the personal histories of participants in future research, so that the psychological basis of these human-animal relationships could be studied.

### 4.6 Conclusion

In conclusion, our study found that individuals in vulnerable situations viewed companion animals as genuine family members and that these animals played an important role in the social support network, particularly as providers of companionship and emotional support. Overall, our findings show that companion animals were more available and, in certain contexts, were even prioritized over human sources, given the number of individuals that relied on them, the range of situations in which they were relied upon and their position in the hierarchy. Importantly, dogs and cats were frequently the sole providers of companionship and emotional comfort, as well as of opportunities for physical closeness and nurturance. Although companion animals fulfilled multiple social needs, our results also indicate that human relationships remain necessary to meet other aspects of social support, such as obtaining guidance (informational support), receiving material or behavioural assistance (instrumental support) and gaining social recognition or approval. The role of companion animals in the social support network is limited when complex cognitive and communicative abilities, practical help or symbolic validation are required. This finding does not reduce their importance as sources of support; instead, it underscores the specificity and uniqueness of their role. Rather than acting as substitutes to human relationships, companion animals constitute a separate and complementary component within the social network, with unique functions and qualities, especially in the emotional domain. The most important quality of companion animals as sources of social support may be their availability, which is particularly valuable for individuals whose social networks are compromised or difficult to access. Safeguarding the bond between vulnerable individuals and their companion animals is fundamental; otherwise, a significant component of their already fragile social networks would be lost.

## Data Availability

The original contributions presented in the study are included in the article/Supplementary material, further inquiries can be directed to the corresponding authors.

## References

[ref1] Applebaum J. W. MacLean E. L. McDonald S. E. (2021). Love, fear, and the human-animal bond: on adversity and multispecies relationships. Compr. Psychoneuroendocrinol. 7:100071. doi: 10.1016/j.cpnec.2021.100071 34485952 PMC8415490

[ref2] Augustine A. Eyssel F. (2024). Motives and risks of self-disclosure to robots versus humans. ACM Trans. Hum.-Robot Interact. 14, 1–44. doi: 10.1145/3700887

[ref3] Beeble M. L. Bybee D. Sullivan C. M. Adams A. E. (2009). Main, mediating, and moderating effects of social support on the well-being of survivors of intimate partner violence across 2 years. J. Consult. Clin. Psychol. 77, 718–729. doi: 10.1037/a0016140, 19634964

[ref4] Bonas S. McNicholas J. Collis G. M. (2000). “Pets in the network of family relationships: an empirical study,” in Companion Animals and Us: Exploring the Relationships Between People and Pets, (New York, NY: Cambridge University Press).

[ref5] Bowen J. Bulbena A. Fatjó J. (2021). The value of companion dogs as a source of social support for their owners: findings from a pre-pandemic representative sample and a convenience sample obtained during the COVID-19 lockdown in Spain. Front. Psych. 12:622060. doi: 10.3389/fpsyt.2021.622060, 33935828 PMC8081030

[ref6] Bowen J. García E. Darder P. Argüelles J. Fatjó J. (2020). The effects of the Spanish COVID-19 lockdown on people, their pets, and the human-animal bond. J. Vet. Behav. 40, 75–91. doi: 10.1016/j.jveb.2020.05.013, 32837452 PMC7292953

[ref7] Chen Y. Hicks A. While A. E. (2014). Loneliness and social support of older people in China: a systematic literature review. Health Soc. Care Community 22, 113–123. doi: 10.1111/hsc.12051, 23714357

[ref8] Cleary M. Thapa D. K. West S. Westman M. Kornhaber R. (2021). Animal abuse in the context of adult intimate partner violence: a systematic review. Aggress. Violent Behav. 61:101676. doi: 10.1016/j.avb.2021.101676

[ref9] Cleary M. Visentin D. Thapa D. K. West S. Raeburn T. Kornhaber R. (2020). The homeless and their animal companions: an integrative review. Admin. Pol. Ment. Health 47, 47–59. doi: 10.1007/s10488-019-00967-6, 31456130

[ref10] Coker A. L. Smith P. H. Thompson M. P. McKeown R. E. Bethea L. Davis K. E. (2002). Social support protects against the negative effects of partner violence on mental health. J. Womens Health Gend. Based Med. 11, 465–476. doi: 10.1089/15246090260137644, 12165164

[ref11] Coker A. L. Watkins K. W. Smith P. H. Brandt H. M. (2003). Social support reduces the impact of partner violence on health: application of structural equation models. Prev. Med. (Baltim). 37, 259–267. doi: 10.1016/S0091-7435(03)00122-1, 12914832

[ref12] Collins N. L. Feeney B. C. (2000). A safe haven: an attachment theory perspective on support seeking and caregiving in intimate relationships. J. Pers. Soc. Psychol. 78, 1053–1073. doi: 10.1037/0022-3514.78.6.1053, 10870908

[ref13] Cryer S. Henderson-Wilson C. Lawson J. (2021). Pawsitive connections: the role of pet support programs and pets on the elderly. Complement. Ther. Clin. Pract. 42:101298. doi: 10.1016/j.ctcp.2020.101298, 33401185

[ref14] Cummings C. Lei Q. Hochberg L. Hones V. Brown M. (2022). Social support and networks among people experiencing chronic homelessness: a systematic review. Am. J. Orthopsychiatry 92, 349–363. doi: 10.1037/ort0000616, 35266727

[ref15] Dickens A. P. Richards S. H. Greaves C. J. Campbell J. L. (2011). Interventions targeting social isolation in older people: a systematic review. BMC Public Health 11:647. doi: 10.1186/1471-2458-11-647, 21843337 PMC3170621

[ref16] Donovan N. J. Blazer D. (2020). Social isolation and loneliness in older adults: review and commentary of a National Academies Report. Am. J. Geriatr. Psychiatry 28, 1233–1244. doi: 10.1016/j.jagp.2020.08.005, 32919873 PMC7437541

[ref17] Dwyer F. Bennett P. C. Coleman G. J. (2006). Development of the Monash dog owner relationship scale (MDORS). Anthrozoös 19, 243–256. doi: 10.2752/089279306785415592

[ref18] El-Alayli A. Lystad A. L. Webb S. R. Hollingsworth S. L. Ciolli J. L. (2006). Reigning cats and dogs: a pet-enhancement bias and its link to pet attachment, pet-self similarity, self-enhancement, and well-being. Basic Appl. Soc. Psych. 28, 131–143. doi: 10.1207/s15324834basp2802_3

[ref19] Epley N. Akalis S. Waytz A. Cacioppo J. T. (2008). Creating social connection through inferential reproduction. Psychol. Sci. 19, 114–120. doi: 10.1111/j.1467-9280.2008.02056.x 18271858

[ref20] Evans-Wilday A. S. Hall S. S. Hogue T. E. Mills D. S. (2018). Self-disclosure with dogs: dog owners’ and non-dog owners’ willingness to disclose emotional topics. Anthrozoös 31, 353–366. doi: 10.1080/08927936.2018.1455467

[ref21] Faraji J. Metz G. A. S. (2021). Aging, social distancing, and COVID-19 risk: Who is more vulnerable and why? Aging Dis. 12, 1624–1643. doi: 10.14336/AD.2021.0319, 34631211 PMC8460299

[ref22] Fatjó J. Bowen J. (2023). “Companion animals in times of crisis,” in The Routledge International Handbook of Human-Animal Interactions and Anthrozoology, (Abingdon: Taylor & Francis).

[ref23] FEDIAF (2024) FEDIAF annual report 2024. Available online at: https://europeanpetfood.org/wp-content/uploads/2024/06/FEDIAF-Annual-Review-2024_Online.pdf (Accessed June 29, 2026).

[ref24] Feeney B. C. Collins N. L. (2015). New look at social support: a theoretical perspective on thriving through relationships. Personal. Soc. Psychol. Rev. 19, 113–147. doi: 10.1177/1088868314544222, 25125368 PMC5480897

[ref25] Fitzgerald A. J. (2007). “They gave me a reason to live”: the protective effects of companion animals on the suicidality of abused women. Hum. Soc. 31, 355–378. doi: 10.1177/016059760703100405

[ref26] Flynn C. P. (2000a). Battered women and their animal companions: symbolic interaction between human and nonhuman animals. Soc. Anim. 8, 99–127. doi: 10.1163/156853000511032

[ref27] Flynn C. P. (2000b). Woman’s best friend. Violence Against Women 6, 162–177. doi: 10.1177/10778010022181778

[ref28] Fraley R. C. Davis K. E. (1997). Attachment formation and transfer in young adults’ close friendships and romantic relationships. Pers. Relat. 4, 131–144. doi: 10.1111/j.1475-6811.1997.tb00135.x

[ref29] Furman W. Buhrmester D. (1985). Children’s perceptions of the personal relationships in their social networks. Dev. Psychol. 21, 1016–1024. doi: 10.1037/0012-1649.21.6.1016

[ref30] Gee N. R. Mueller M. K. (2019). A systematic review of research on pet ownership and animal interactions among older adults. Anthrozoös 32, 183–207. doi: 10.1080/08927936.2019.1569903

[ref31] Gillath O. Karantzas G. C. Fraley R. C. (2016). “What is an attachment relationship?” in Adult Attachment, (San Diego, CA: Academic Press).

[ref32] Gravrok J. Bendrups D. Howell T. Bennett P. C. (2020). ‘Thriving through relationships’ as a useful adjunct to existing theoretical frameworks used in human-companion dog interaction literature. Hum. Anim. Interact. Bull. 8, 3–22. doi: 10.1079/hai.2020.0014, 36007395

[ref33] HABRI (2023) The universal human-animal bond. Available online at: https://habri.org/assets/uploads/HABRI-Zoetis-International-HAB-Survey-Full-Presentation-July-2023.pdf (Accessed June 29, 2026).

[ref34] Hageman T. O. Langenderfer-Magruder L. Greene T. Williams J. H. Mary J. McDonald S. E. . (2018). Intimate partner violence survivors and pets: exploring practitioners’ experiences in addressing client needs. Fam. Soc. 99, 134–145. doi: 10.1177/1044389418767836

[ref35] Hardie S. Mai D. L. Howell T. J. (2023). Social support and wellbeing in cat and dog owners, and the moderating influence of pet–owner relationship quality. Anthrozoös 36, 891–907. doi: 10.1080/08927936.2023.2182029

[ref36] Hazan C. Hutt M. Sturgeon J. Bricker T. (1991). The Process of Relinquishing Parents as Attachment Figures. Seattle, WA: Biennial Meeting of the Society for Research in Child Development.

[ref37] Hazan C. Zeifman D. (1994). “Sex and the psychological tether,” in Attachment Processes in Adulthood, eds. Bartholomew K. Perlman D. (London: Jessica Kingsley Publishers).

[ref38] Holland K. E. Mead R. Casey R. A. Upjohn M. M. Christley R. M. (2022). Why do people want dogs? A mixed-methods study of motivations for dog Acquisition in the United Kingdom. Front. Vet. Sci. 9:877950. doi: 10.3389/fvets.2022.877950, 35619602 PMC9127952

[ref39] Howell T. J. Bowen J. Fatjó J. Calvo P. Holloway A. Bennett P. C. (2017). Development of the cat-owner relationship scale (CORS). Behav. Process. 141, 305–315. doi: 10.1016/j.beproc.2017.02.024, 28279780

[ref40] Hughes M. J. Verreynne M.-L. Harpur P. Pachana N. A. (2020). Companion animals and health in older populations: a systematic review. Clin. Gerontol. 43, 365–377. doi: 10.1080/07317115.2019.1650863, 31423915

[ref41] Irvine L. Cilia L. (2017). More-than-human families: pets, people, and practices in multispecies households. Sociol. Compass 11:e12455. doi: 10.1111/soc4.12455

[ref42] Jagoe A. Serpell J. A. (1996). Owner characteristics and interactions and the prevalence of canine behaviour problems. Appl. Anim. Behav. Sci. 47, 31–42. doi: 10.1016/0168-1591(95)01008-4

[ref43] Katerndahl D. Burge S. Ferrer R. Becho J. Wood R. (2013). Differences in social network structure and support among women in violent relationships. J. Interpers. Violence 28, 1948–1964. doi: 10.1177/0886260512469103, 23262818

[ref44] Kerman N. Gran-Ruaz S. Lem M. (2019). Pet ownership and homelessness: a scoping review. J. Soc. Distress Homeless 28, 106–114. doi: 10.1080/10530789.2019.1650325

[ref45] Krause-Parello C. A. Gulick E. E. Basin B. (2019). Loneliness, depression, and physical activity in older adults: the therapeutic role of human–animal interactions. Anthrozoös 32, 239–254. doi: 10.1080/08927936.2019.1569906

[ref46] Kurdek L. A. (2008). Pet dogs as attachment figures. J. Soc. Pers. Relat. 25, 247–266. doi: 10.1177/0265407507087958

[ref47] Kurdek L. A. (2009a). Pet dogs as attachment figures for adult owners. J. Fam. Psychol. 23, 439–446. doi: 10.1037/a0014979, 19685978

[ref48] Kurdek L. A. (2009b). Young adults’ attachment to pet dogs: findings from open-ended methods. Anthrozoös 22, 359–369. doi: 10.2752/089279309X12538695316149

[ref49] Labrecque J. Walsh C. A. (2011). Homeless women’s voices on incorporating companion animals into shelter services. Anthrozoös 24, 79–95. doi: 10.2752/175303711X12923300467447

[ref50] Liu H. Lin J. Lin W. (2024). Cognitive mechanisms and neurological foundations of companion animals’ role in enhancing human psychological well-being. Front. Psychol. 15:1354220. doi: 10.3389/fpsyg.2024.1354220, 38721326 PMC11076790

[ref51] Mah J. Rockwood K. Stevens S. Keefe J. Andrew M. (2022). Do interventions reducing social vulnerability improve health in community dwelling older adults? A systematic review. Clin. Interv. Aging 17, 447–465. doi: 10.2147/CIA.S349836 35431543 PMC9012306

[ref52] McConnell A. R. Brown C. M. Shoda T. M. Stayton L. E. Martin C. E. (2011). Friends with benefits: on the positive consequences of pet ownership. J. Pers. Soc. Psychol. 101, 1239–1252. doi: 10.1037/a0024506, 21728449

[ref53] McConnell A. R. Lloyd E. P. Buchanan T. M. (2016). “Animals as friends: social psychological implications of human–pet relationships,” in The Psychology of Friendship, eds. Hojjat M. Moyer A. (Oxford: Oxford University Press).

[ref54] McConnell A. R. Paige Lloyd E. Humphrey B. T. (2019). We are family: viewing pets as family members improves wellbeing. Anthrozoös 32, 459–470. doi: 10.1080/08927936.2019.1621516

[ref55] McCosker L. K. Maujean A. Hill N. Downes M. J. (2024). Services and interventions for people who are homeless with companion animals (pets): a systematic review. J. Soc. Distress Homelessness 33, 583–593. doi: 10.1080/10530789.2023.2205188

[ref56] Meehan M. Massavelli B. Pachana N. (2017). Using attachment theory and social support theory to examine and measure pets as sources of social support and attachment figures. Anthrozoös 30, 273–289. doi: 10.1080/08927936.2017.1311050

[ref57] Newberry M. (2017). Pets in danger: exploring the link between domestic violence and animal abuse. Aggress. Violent Behav. 34, 273–281. doi: 10.1016/j.avb.2016.11.007

[ref58] Nolet A.-M. Morselli C. Cousineau M.-M. (2021). The social network of victims of domestic violence: a network-based intervention model to improve relational autonomy. Violence Against Women 27, 1630–1654. doi: 10.1177/1077801220947169, 32814488

[ref59] Petrovich J. C. Cronley C. C. (2015). Deep in the heart of Texas: a phenomenological exploration of unsheltered homelessness. Am. J. Orthopsychiatry 85, 315–323. doi: 10.1037/ort0000043, 25602352

[ref60] Posit Team (2025) RStudio: integrated development environment for R. Available online at: http://www.posit.co/ (Accessed June 29, 2026).

[ref61] Přibylová L. Součková M. Vostrá-Vydrová H. Glenk L. M. (2025). Perceived relationships and the costs and benefits of dog ownership in Czech homeless and non-homeless people. Anthrozoös 38, 371–387. doi: 10.1080/08927936.2024.2425176

[ref62] Reniers P. W. A. Declercq I. J. N. Hediger K. Enders-Slegers M.-J. Gerritsen D. L. Leontjevas R. (2023). The role of pets in the support systems of community-dwelling older adults: a qualitative systematic review. Aging Ment. Health 27, 1377–1387. doi: 10.1080/13607863.2022.2141196, 36325924

[ref63] Rowe A. C. Carnelley K. B. (2005). Preliminary support for the use of a hierarchical mapping technique to examine attachment networks. Pers. Relat. 12, 499–519. doi: 10.1111/j.1475-6811.2005.00128.x

[ref64] Scanlon L. Hobson-West P. Cobb K. McBride A. Stavisky J. (2021). Homeless people and their dogs: exploring the nature and impact of the human–companion animal bond. Anthrozoös 34, 77–92. doi: 10.1080/08927936.2021.1878683

[ref65] Sudbury-Riley L. (2024). COVID companions: exploring pets as social support. Sociol. Heal. Illn. 46, 1923–1941. doi: 10.1111/1467-9566.13820, 39073135

[ref66] Tancredy C. M. Fraley R. C. (2006). The nature of adult twin relationships: an attachment-theoretical perspective. J. Pers. Soc. Psychol. 90, 78–93. doi: 10.1037/0022-3514.90.1.78, 16448311

[ref67] The Jamovi Project (2025) Jamovi. Avaialble online at: https://www.jamovi.org (Accessed June 29, 2026).

[ref68] Thoits P. A. (2011). Mechanisms linking social ties and support to physical and mental health. J. Health Soc. Behav. 52, 145–161. doi: 10.1177/0022146510395592, 21673143

[ref69] Thomas D. R. Hodges I. D. (2026). A new taxonomy of social support: clarifying supportive behaviours and measures. J. Health Psychol. 31, 3033–3049. doi: 10.1177/13591053251412946, 41581206 PMC13287499

[ref70] Toohey A. M. Hewson J. A. Adams C. L. Rock M. J. (2017). When ‘places’ include pets: broadening the scope of relational approaches to promoting aging-in-place. J. Sociol. Soc. Welf. 44, 119–146. doi: 10.15453/0191-5096.3875

[ref71] Trinke S. J. Bartholomew K. (1997). Hierarchies of attachment relationships in young adulthood. J. Soc. Pers. Relat. 14, 603–625. doi: 10.1177/0265407597145002

[ref72] Turcsán B. Ujfalussy D. J. Kerepesi A. Miklósi Á. Kubinyi E. (2025). Similarities and differences between dog – human and human – human relationships. Sci. Rep. 15:11871. doi: 10.1038/s41598-025-95515-8, 40258899 PMC12012045

[ref73] Watson J. Crawley J. Kane D. (2016). Social exclusion, health and hidden homelessness. Public Health 139, 96–102. doi: 10.1016/j.puhe.2016.05.017, 27340041

[ref74] Weiss R. S. (1974). “The provisions of social relationships,” in Doing unto Others: Joining, Molding, Conforming, Helping, Loving, ed. Rubin Z. (Englewood Cliffs, NJ: Prentice-Hall).

[ref75] Wilson D. M. Harris A. Hollis V. Mohankumar D. (2011). Upstream thinking and health promotion planning for older adults at risk of social isolation. Int. J. Older People Nurs. 6, 282–288. doi: 10.1111/j.1748-3743.2010.00259.x, 21631882

[ref76] Wood L. Martin K. Christian H. Nathan A. Lauritsen C. Houghton S. . (2015). The pet factor - companion animals as a conduit for getting to know people, friendship formation and social support. PLoS One 10:e0122085. doi: 10.1371/journal.pone.0122085, 25924013 PMC4414420

[ref77] Zugazaga C. B. (2008). Understanding social support of the homeless: a comparison of single men, single women, and women with children. Fam. Soc. 89, 447–455. doi: 10.1606/1044-3894.3770

